# IMMUNOLOGICAL EVALUATION OF PATIENTS WITH TYPE 2 DIABETES MELLITUS SUBMITTED TO METABOLIC SURGERY

**DOI:** 10.1590/S0102-6720201500040012

**Published:** 2015

**Authors:** Marisa de Carvalho BORGES, Guilherme Azevedo TERRA, Tharsus Dias TAKEUTI, Betânia Maria RIBEIRO, Alex Augusto SILVA, Júverson Alves TERRA-JÚNIOR, Virmondes RODRIGUES-JÚNIOR, Eduardo CREMA

**Affiliations:** 1 Department of Immunology; 2 Department of Surgery, Federal University of Triângulo Mineiro, Uberaba, MG, Brazil

**Keywords:** Cytokines, Diabetes mellitus, Surgery

## Abstract

**Background:**

Immunological and inflammatory mechanisms play a key role in the development and progression of type 2 diabetes mellitus.

**Aim:**

To raise the hypothesis that alterations in immunological parameters occur after duodenojejunal bypass surgery combined with ileal interposition without gastrectomy, and influences the insulin metabolism of betacells.

**Methods:**

Seventeen patients with type 2 diabetes mellitus under clinical management were submitted to surgery and blood samples were collected before and six months after surgery for evaluation of the serum profile of proinflammatory (IFN-γ, TNF-α, IL-17A) and anti-inflammatory cytokines (IL-4, IL-10). In addition, anthropometric measures, glucose levels and insulin use were evaluated in each patient.

**Results:**

No changes in the expression pattern of proinflammatory cytokines were observed before and after surgery. In contrast, there was a significant decrease in IL-10 expression, which coincided with a reduction in the daily insulin dose, glycemic index, and BMI of the patients. Early presentation of food to the ileum may have induced the production of incretins such as GLP-1 and PYY which, together with glycemic control, contributed to weight loss, diabetes remission and the consequent good surgical prognosis of these patients. In addition, the control of metabolic syndrome was responsible for the reduction of IL-10 expression in these patients.

**Conclusion:**

These findings suggest the presence of low-grade inflammation in these patients during the postoperative period, certainly as a result of adequate glycemic control and absence of obesity, contributing to a good outcome of surgery.

## Abstract (pt)

***Racional*:**

Mecanismos imunológicos e inflamatórios desempenham papel-chave no desenvolvimento e progressão do diabete melito tipo 2.

***Objetivo*:**

Levantar a hipótese de que alterações nos parâmetros imunológicos ocorrem após operação duodenojejunal combinada com interposição ileal sem gastrectomia, e influenciam o metabolismo da insulina das células beta.

***Métodos*:**

Dezessete pacientes com diabete melito tipo 2 sob manejo clínico foram submetidos à cirurgia e amostras de sangue foram coletadas antes e seis meses após para avaliação do perfil de sorológico de citocinas pró-inflamatórias (IFN-γ, TNF-α, IL-17A) e anti-inflamatórias(IL-4, IL-10). Além disso, parâmetros antropométricos, glicemia e uso de insulina foram avaliados em cada paciente.

***Resultados*:**

Não ocorreram alterações no padrão de expressão de citocinas pró-inflamatórias observadas antes e depois da operação. Em contraste, houve diminuição significativa na expressão de IL-10, que coincide com redução da dose diária de insulina, com o controle glicêmico e redução do IMC dos pacientes. Apresentação precoce de alimentos para o íleo pode ter induzido a produção das incretinas tais como GLP-1 e PYY, que, juntamente com o controle da glicemia, contribuíram para a perda de peso, remissão do diabete e o bom prognóstico consequente cirúrgico. Além disso, o controle de síndrome metabólica foi responsável pela redução da expressão de IL-10 nestes doentes.

***Conclusão*:**

Baixo grau de inflamação estava presente nesses pacientes no pós-operatório, certamente pelo adequado controle glicêmico e ausência de obesidade, o que contribuiu para bom resultado cirúrgico.

## INTRODUCTION

Diabetes mellitus is a chronic disease characterized by relative or absolute insulin deficiency and consequent glucose intolerance. The World Health Organization estimates that about 240 million people worldwide have diabetes and this figure is likely to increase to more than 50% by 2025, with 380 million people suffering from this disease^13^.

Immunological and inflammatory mechanisms play a key role in the development and progression of type 2 diabetes mellitus^16^. Herder *et al* .^11 ^showed that elevated TGF-β1 concentrations indicate an increased risk of progression to type 2 diabetes and that subclinical inflammation leads to insulin resistance and pancreatic beta-cell dysfunction. According to Kopp *et al* .^12^^,^ elevated levels of C-reactive protein and IL-6 indicate chronic subclinical inflammation and are associated with metabolic syndrome and cardiovascular diseases. Taken together, these results suggest a bidirectional relationship between insulin resistance and inflammation, i.e., any chronic inflammatory process induces insulin resistance which, in turn, enhances the inflammatory process^6^.

A variety of treatment options exist for the management of insulin resistance, including a multidisciplinary clinical approach designed to promote weight loss, pharmacological therapies, and bariatric and metabolic surgical techniques^15^^,^ 17 ^-^ 18. Ileal transposition involves the removal of a segment of the distal ileum and its insertion into the proximal small intestine, a procedure that promotes early satiety and exerts beneficial effects on glucose metabolism and weight loss. These effects can probably be attributed to the stimulation of incretins such as GLP-1 and PYY, increasing short- and medium-term insulin sensitivity^8^. Surgical treatment consisting of duodenojejunal bypass with or without ileal segment interposition has been shown to permit clinical control of patients with type 2 diabetes without the need for insulin or oral hypoglycemic agents^7^^,^ 3.

There are no studies in the literature investigating the expression of proinflammatory (IFN-γ, TNF-α, IL-17A) and anti-inflammatory (IL-4, IL-10) cytokines in serum of patients with type 2 diabetes mellitus submitted to duodenojejunal bypass surgery with ileal interposition without gastric resection. 

The present study raises the hypothesis that alterations in immunological parameters, expressed as the production of cytokines in serum, occur after ileal interposition and influence the insulin metabolism of beta cells.

## METHODS

A prospective, cross-sectional study was conducted at the Disciplines of Digestive Tract Surgery and Immunology, Universidade Federal do Triângulo Mineiro (UFTM), Uberaba, MG, Brazil. The study was approved by the Ethics Committee of UFTM (protocol No. 1686) and the patients signed a free informed consent form. The patients were selected between January 2009 and January 2010.

Seventeen adults, aged 21 to 60 years, with type 2 diabetes mellitus and a body mass index (BMI) of 22 to 34 kg/m^2^ were selected by intentional sampling.

**FIGURE 1:**
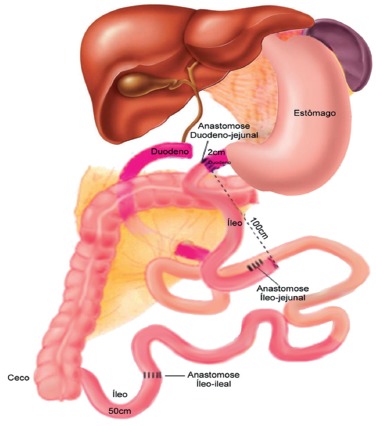
- Scheme of the surgery: duodenojejunal exclusion with ileal interposition without gastrectomy

Patients with severe heart disease, patients presenting an elevated surgical risk (ASA IV), diabetic patients diagnosed less than three years ago, patients with type 1 diabetes and/or other endocrine abnormalities, with chronic inflammatory disease and refusal to undergo the treatment proposed were excluded. All volunteers were submitted to duodenojejunal bypass with interposition of an ileal segment without gastric resection. The procedure consists of interposition of an ileal segment measuring approximately 100 cm. This segment is transposed and anastomosed to the duodenum 2 cm from the pylorus and to the jejunum 70 cm from duodenojejunal angle, thus excluding 100 cm of the duodenojejunal segment (Figure 1).

Blood samples were collected from all patients 24 h before the surgical procedure and six months after surgery after a 12 h overnight fast. The blood sample was centrifuged immediately at 5.000 rpm and the supernatant was aspirated and stored in 1.5-ml sterile plastic tubes at -70°C.

Glucose was measured by a colorimetric enzymatic method using commercially available kits. Serum cytokines (IFN-γ, TNF-α, IL-17A, IL-4, and IL-10) were determined by enzyme-linked immunosorbent assay (ELISA) using commerciallyavailable monoclonal antibodies.

High-affinity 96-well plates (Nunc, Denmark) were sensitized with the specific monoclonal antibodies. Lanes 1 and 2 of each plate received 100 µl of serial dilutions (1:2) of the recombinant cytokine standard in phosphate-buffered saline (PBS) containing 2% human serum albumin (BSA). No cytokine or serum was added to the wells corresponding to the reaction blank. Next, 100 µl/well of serum containing the cytokine to be measured was added to the other lanes. The plates were incubated for 18 h at 4°C and then washed six times in PBS-Tween 20 (PBS-T). Next, 100 µl/well of the biotinylated anti-cytokine antibody diluted 1:1,000 in PBS-1% BSA was added. The plates were incubated for 2 h at 37°C and washed again six times in PBS-T. After this step, 100 µl/well alkaline phosphatase-labeled streptavidin, diluted 1:1,000 in PBS-1% BSA, was added and the plates were incubated for 1 h. Next, the plates were washed six times in PBS-T and the reaction was developed by the addition of 100 µl/well dinitrophenyl phosphate as substrate. Absorbance was read in an automated ELISA reader (Bio-Rad 2550 EIA Reader) and the results were determined as the difference in absorbance at 405 and 490 nm (Abs 405 - Abs 409). Serum cytokine concentration was calculated by linear regression from the standard curve of the recombinant molecule and is expressed as pg/ml. 

The Kolmogorov-Smirnov test was used to determine whether the data were normally distributed. Parametric data were compared by the Student t-test and non-parametric data by the Wilcoxon test. Correlations were evaluated using Pearson's and Spearman's correlation coefficients. Differences were considered to be significant when p<0.05. Statistical analysis was performed using the Microsoft Excel 2010, GraphPad Prism 5.0,and SPSS 16.0 programs.

## RESULTS

Seventeen patients with a diagnosis of type 2 diabetes mellitus, who had used insulin for at least two years and were followed up at the outpatient service of the University Hospital of UFTM, participated in the study. The mean age of the patients was 55.4 (±8.66) years (34-68). Ten (58.8%) were females and seven (41.2%) males. 

The BMI was used for the evaluation of body weight. Two (11.8%) patients were normal weight (BMI: 18 to 24.99 kg/m^2^), 10 (59%) were overweight (BMI: 25 to 29.99 kg/m^2^), and five (19.2%) had obesity grade I (BMI: 30 to 34.99 kg/m^2^). The mean BMI was 29.52 kg/m^2^ (±2.91).

Preoperative glycemia was elevated in all patients, with a mean level of 207.65 (±5.3) mg/dl (116.8-322.5). The mean insulin dose used by these patients before surgery was 60.8 (±29.9) U (27-150), demonstrating the metabolic decompensation of these patients, with no response to clinical management, even with high insulin intake.

Analysis of the preoperative cytokine profile showed no significant levels of proinflammatory cytokines (IFN-γ, TNF-α, or IL-17A), with the observation of sporadic positive results in isolated patients. In contrast, marked expression of IL-10 was observed in the patients before surgery (111.85±147.48 pg/ml). No significant expression of IL-4 was detected in the group studied. 

Postoperative follow-up (six months after surgery) showed a significant BMI reduction in the patients, with a mean of 27.32 (±3.46) (p=0.0032). This weight loss was accompanied by a significant decline in fasting glycemia (135.7±32.75 mg/dl, range: 76.6 to 196.9 mg/dl) (p<0.0001). In addition, there was a reduction in the daily doses of insulin used by the patients, with a mean daily dose of 11.8 (±16.7) U (0-44) (p<0.001). Nine (53%) patients discontinued insulin therapy within the first six months. These patients were able to maintain low blood glucose levels only with diet combined or not with oral hypoglycemic drugs. 

**FIGURE 2:**
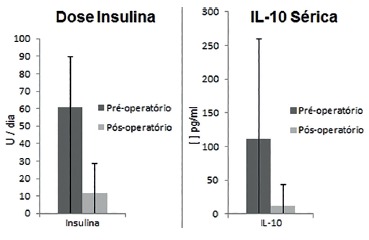
- Pre- and postoperative insulin dose (a) and serum IL-10 (b) in diabetic patients submitted to duodenojejunal bypass with ileal interposition without gastrectomy. Values are the mean and standard deviation. A significant reduction in insulin and IL-10 was observed: a, p<0.001; b, p=0.006.

Analysis of the postoperative cytokine profile again showed no significant presence of proinflammatory cytokines (IFN-γ, TNF-α, IL-17A) or IL-4. However, a significant decrease was observed in the expression of IL-10 (11.62 ±32.26 pg/ml, p=0.003) (Figure 2). This decline was correlated with a decrease in the insulin dose used by the patients after surgery (r=0.53 and p=0.06) 

## DISCUSSION

Chronic hyperglycemia is due mainly to an increase in glycated proteins, which stimulate the production of cytokines related to the long-term complications of diabetes such as increased susceptibility to infection and impaired wound healing^1^.

Proinflammatory cytokines such as IL-1β, IL-6 and TNF-α have been reported to play a critical role in insulin resistance and in the pathogenesis of type 2 diabetes mellitus^2^. These cytokines exert cytotoxic, cytostatic (inhibition of the synthesis and secretion of insulin), or cytocidal action on the pancreatic islands, stimulating the production of nitric oxide. Together with C-reactive protein, these cytokines can induce an acute inflammatory process^5^.

In the present study, no significant expression of proinflammatory cytokines (TNF-α, IFN-γ, or IL-17A) was observed before metabolic surgery. However, preoperative expression of IL-10 was detected in 14 of the 17 patients, which may have inhibited the expression of proinflammatory cytokines.

The use of insulin for more than two years by the patients studied here may have contributed to the high preoperative levels of IL-10. According to Frankie*et al* .^9^, insulin exerts an anti-inflammatory effect by acting on the glycemic control of patients with type 2 diabetes mellitus. Geerlings *et al* .^10^observed an increased expression of IL-10 in patients with type 2 diabetes mellitus who achieved adequate metabolic control.

IL-10 has been shown to regulate Th1 immune responses, but the biological activity of this cytokine appears to be more complex and there is evidence of proinflammatory effects^14^. Choi *et al* .^4^ found higher IL-10 levels in subjects without metabolic syndrome when compared to patients with metabolic syndrome.

A significant decline in IL-10 expression was observed six months after surgery. This finding might be attributed to the fact that most patients no longer used insulin or oral hypoglycemic agents. 

Metabolic surgery performed in the present study yielded satisfactory results, with improvement of glucose metabolism and control of cholesterol and triglyceride levels. No significant expression of proinflammatory or anti-inflammatory (IL-4) cytokines was observed during the postoperative period.

The present results might be explained by the mechanism of the distal ileum which activated the production of GLP-1 and/or peptides in the distal intestine, promoting improved clinical control of type 2 diabetes mellitus. 

 However, further studies are needed to identify new inflammatory markers that interfere with insulin metabolism of beta cells before and after metabolic surgery in order to improve the clinical and/or surgical treatment of these patients. 

## CONCLUSION

These findings suggest the presence of low-grade inflammation in these patients during the postoperative period, certainly as a result of adequate glycemic control and absence of obesity, contributing to a good outcome of surgery.
